# Automatic Evaluations and Exercising: Systematic Review and Implications for Future Research

**DOI:** 10.3389/fpsyg.2017.02103

**Published:** 2017-12-01

**Authors:** Michaela Schinkoeth, Franziska Antoniewicz

**Affiliations:** Sport and Exercise Psychology, Department of Sport and Health Sciences, University of Potsdam, Potsdam, Germany

**Keywords:** automatic evaluation, exercise, associative, dual-process, implicit attitude, affective

## Abstract

The general purpose of this systematic review was to summarize, structure and evaluate the findings on automatic evaluations of exercising. Studies were eligible for inclusion if they reported measuring automatic evaluations of exercising with an implicit measure and assessed some kind of exercise variable. Fourteen nonexperimental and six experimental studies (out of a total *N* = 1,928) were identified and rated by two independent reviewers. The main study characteristics were extracted and the grade of evidence for each study evaluated. First, results revealed a large heterogeneity in the applied measures to assess automatic evaluations of exercising and the exercise variables. Generally, small to large-sized significant relations between automatic evaluations of exercising and exercise variables were identified in the vast majority of studies. The review offers a systematization of the various examined exercise variables and prompts to differentiate more carefully between actually observed exercise behavior (proximal exercise indicator) and associated physiological or psychological variables (distal exercise indicator). Second, a lack of transparent reported reflections on the differing theoretical basis leading to the use of specific implicit measures was observed. Implicit measures should be applied purposefully, taking into consideration the individual advantages or disadvantages of the measures. Third, 12 studies were rated as providing first-grade evidence (lowest grade of evidence), five represent second-grade and three were rated as third-grade evidence. There is a dramatic lack of experimental studies, which are essential for illustrating the cause-effect relation between automatic evaluations of exercising and exercise and investigating under which conditions automatic evaluations of exercising influence behavior. Conclusions about the necessity of exercise interventions targeted at the alteration of automatic evaluations of exercising should therefore not be drawn too hastily.

## Introduction

Health behavior is not solely based on the reflective processing of information. It is also driven by automatic processes (Kahneman, 2003). This duality in the processing of information is theoretically described in dual-process theories (e.g., Strack and Deutsch, 2004; Gawronski and Bodenhausen, 2006; Evans and Stanovich, 2013) that distinguish between reflective (also termed “explicit, propositional”) and automatic (also termed “implicit, associative”) processing of information. Reflective processes are characterized by conscious deliberation on available information. Resulting reflective evaluations can be cognitively (e.g., “exercise is healthy”) or affectively (e.g., “exercise is fun”) shaped. *Automatic evaluations* as an output of automatic processes represent the affective, spontaneous and often unconscious reaction to a present stimulus or event. The reaction arises from the (often previously learned) mental associations between a target concept (e.g., exercise) and affective attributes (e.g., pleasant, tiring) and is based on the mental representation of one's core affective reactions (Ekkekakis, 2013) to the stimulus or the event. According to dual-process theories, behavioral decisions are the result of interactions between both processes and thus between automatic and reflective evaluations (Strack and Deutsch, 2004; Gawronski and Bodenhausen, 2006). Automatic evaluations can be, but are not necessarily, in agreement with the reflective evaluations of a given object (e.g., Friese et al., 2011). One often referred to dual-process model, the associative-propositional model (APE), offers theoretical assumptions about mutual influences of automatic and reflective evaluations (Gawronski and Bodenhausen, 2011). For example, when automatic and reflective evaluations are inconsistent the initial reflective evaluation can be resolved in order to avoid aversive feelings. Furthermore, dual-process theories provide a framework for possible relations between automatic evaluations and exercise behavior. For example, according to the reflective-impulsive model (RIM, Strack and Deutsch, 2004) automatically activated associations trigger behavioral schemata that contain a motivational orientation toward approaching or avoiding the respective behavior. Automatic evaluations can thus for example help in understanding the paradoxical phenomenon of nonexercising. Individuals often know that exercising is good for them (reflective cognitive evaluation) but might automatically feel bad about it and thus decide against it (Bluemke et al., 2010).

Theorizing on health behavior and health behavior change (e.g., Deci and Ryan, 1980; Prochaska and DiClemente, 1984; Ajzen, 1985; Bandura, 1986) focused for more than half a century on reflective processes and almost completely neglected the potential relevance of automatic antecedents of behavior. Major social-cognitive theories and the consequential health interventions assume that by providing information, intentions are changed, which will lead to respective changes in behavior. However, meta-analysis revealed only a weak relation between altered intentions and exercise behavior (*d* = 0.15; Rhodes and Dickau, 2012) and concluded that this relation has very low practical value. Others commented that moving away from the underlying social-cognitive “information processing paradigm” (Ekkekakis, 2017), which is based on the meta-theoretical assumption that human beings act as rational information collectors who reflectively arrive at their decisions, could offer the opportunity to re-establish a new way of looking at a well-known phenomenon (i.e., why a large proportion of people fail to adapt to related health behavior interventions).

Since the effects of the mentioned interventions are rather sobering, Ekkekakis (2017, p. 86) anticipated that exercise psychology might be “undergoing a transition to dual-process theoretical models for conceptualizing the mechanisms that shape behavioral decisions about participation or nonparticipation in exercise and physical activity” and proposed that exercise psychology is already within a meta-theoretical crisis. Marteau et al. (2012) offer a more applied perspective when acknowledging that health interventions that additionally target “automatic bases of behaviors may be more effective” (p. 1492) than interventions that address reflective processes solely. Following this line of argumentation Conroy and Berry (2017) consider automatic evaluations of exercise and physical activity as “potentially modifiable targets” (p. 236) for specific interventions.

Taking the described—possible—meta-theoretical transition into account, the first necessary step in order to better understand the potential of automatic process theorizing for exercise psychology is the systematic summary and evaluation of related, so far accumulated, empirical evidence.

A growing body of literature already underlines the relevance of automatic evaluations for health behaviors (Friese et al., 2011; Hagger, 2016) such as eating (Friese et al., 2008), smoking (Payne et al., 2007), alcohol intake (Houben and Wiers, 2008), and physical activity (Conroy et al., 2010). There is ample evidence that automatic evaluations are related to exercise decisions and exercise behavior as well (e.g., Bluemke et al., 2010; Brand and Schweizer, 2015). Until now there has been no systematic review on automatic evaluations of exercising (AEE) and relations to exercise behavior. This lack of systematically gained, summarized and evaluated findings hinders progress in the field.

### The focus of this review

This review focuses research on AEE and exercise. AEE can be related to physiological or psychological correlates of actual exercise behavior (e.g., exercise-related decisions) or exercise behavior itself (e.g., exercise amounts, exercise adherence). For the purpose of this review, we will use henceforth the umbrella term *exercise indicators* when referring to the various different measures of exercise-related constructs.

There are a variety of different terms when speaking about AEE. While some researchers seem to prefer the term “implicit attitudes” (e.g., Calitri et al., 2009; Markland et al., 2015), others have used the term “affective associations” (e.g., Sala et al., 2016). While the synonymous use of these terms is generally non-admissible, in this review, we checked thoroughly that all included studies targeted the affective core of the assessed evaluations in order to subsume the identified evaluations as AEE.

Literature on automatic evaluations describes various implicit measures for the assessment of automatic evaluations (for an overview see Gawronski and De Houwer, 2014). Prominent examples of these implicit measures are the Implicit Association Test (IAT; Greenwald et al., 1998) and the Evaluative Priming Task (EP; Fazio et al., 1986). One common aspect of these methods is avoiding verbal or written self-reports (e.g., questionnaires) but rather employing computerized tools that indirectly assess AEE through within-person response time latencies, interpersonal reaction time differences and error rates. Resulting scores are interpreted as indices of the strength of associations between a target concept (“exercise”) and affective attributes (valence). This review includes an overview of the most commonly used methods to measure AEE. Their expedient use is evaluated and implications for future research discussed.

The aim of this systematic review is to summarize and structure existing findings on AEE and relations to exercise indicators. In order to appraise the magnitude of the impact of AEE on exercise indicators, effect sizes will be extracted from the identified studies where possible.

## Methods

### Search strategy

A systematic literature search was carried out in May 2017 and conducted according to the PRISMA guidelines (Liberati et al., 2009). The following databases were used: PsycINFO, PSYNDEX, PsycARTICLES, SPORTDiscus and PubMed (see footnote^1^ for the used search terms). The search included the articles' titles, abstracts and keywords. We exclusively searched for peer-reviewed articles. There was no restriction on year of publication. Additionally, the reference lists of the identified articles were screened for further articles.

### Eligibility criteria and study selection

The authors executed the initial screening of all retrieved studies on the basis of titles and abstracts independently from each other. The following inclusion and exclusion criteria were applied. Only original study reports in English or German were considered for integration in this review. Studies had to include an implicit assessment of AEE and some kind of exercise indicator to be eligible. Due to the use of diverse terms when referring to AEE we thoroughly checked if the assessed automatic evaluations in the identified studies fit to the described definition of automatic (also called “spontaneous,” “associative,” “uncontrolled,” or “implicit”) affective evaluations of exercising. Additionally, studies that did not examine exercise indicators were excluded. Exercise was defined as a “subset of physical activity that is planned, structured, and repetitive” (Caspersen et al., 1985, p. 126). Selected studies had to include human participants, be interventional or observational and gather cross-sectional or longitudinal data. Structured consultations between the two authors were carried out to eliminate disagreement regarding the eligibility of contentious studies. Afterwards, the two authors independently performed full-text reviews (based on the described inclusion and exclusion criteria) of all (pre-)selected articles. Finally, 19 records were included in further evaluation.

Referring to the proceeding of Rebar et al. (2016a), all studies' grades of evidence based on study design were evaluated. Cross-sectional studies were considered to provide first-grade evidence, prospective and longitudinal studies were taken to represent the second grade of evidence and experimental studies were considered to provide third-grade evidence. Ratings could have been lowered when risks of bias were present. Risks of bias were classified on study level following the guidelines from the Qualitative Assessment Tool for Quantitative Studies (Effective Public Health Practice Project, 2009). More specifically, the following sources of bias were evaluated: selection bias (when individuals selected to take part in the study were not likely to be representative of the target population), inadequate blinding of study participants (e.g., high risk of bias when participants were aware of the research question), unreliable data collection methods (e.g., high risk of bias when data relied on self-reports only), existence of confounders (e.g., high risk of bias when important differences between groups were present prior to manipulations), and insufficient reports of withdrawals and dropouts (for further information see Effective Public Health Practice Project, 2009). Only one rating was lowered from second to first-grade of evidence (Craeynest et al., 2008). Two cross-sectional studies showed risks of biases too (Berry et al., 2011; Sala et al., 2016). Since both studies were already rated as providing first-grade of evidence the rating was not adjusted.

### Data extraction and synthesis

We summarized the specific characteristics of the studies separately for nonexperimental (see Table 1) and experimental (see Table 2) studies. After coding each study, great heterogeneity in the applied implicit and outcome measures became apparent. Due to the incomparability of the studies it was decided that it was impossible to conduct a meta-analysis (Ioannidis et al., 2008), so we carried out a systematic review instead. Nevertheless, we report significant effect sizes when available in the included studies. For reasons of clarity and comprehensibility we use Cohen's *d* as the effect size that expresses the degree of difference between groups (or means). Originally reported effect sizes (e.g., $\eta_{part}^{2}$ or η^2^) were transformed into Cohen's *d* where necessary (Lenhard and Lenhard, 2016). To express the degree of association between variables, the correlation coefficient *r* or the unstandardized and standardized regression coefficients *b* and β were used. The determination coefficient *R*^2^ indicates the proportion of variance in one variable that is explained by another variable.

**Table 1:** Nonexperimental studies investigating automatic evaluations of exercising and exercise indicators (*k* = 14).

	**Study**	**Participants**	**Study design**	**Main aim**	**AEE measure**	**Main proximal and distal exercise indicators**	**Main findings concerning AEE**	**Grade of evidence rating**
1	Antoniewicz and Brand, 2014	72 graduate sport and exercise students, *M* = 26.00 (*SD* = 9.03)	Cross-sectional	Examine the relation between AEE and preferred exercise-setting	AMP with subliminal presented fitness center pictures	**Proximal**: preferred exercise- setting (center exerciser, comparison group); reflective affective evaluations	A MANOVA indicated a significant group effect, and a test of between-subject differences revealed significant group effects on AEE (*d* = 0.59) and reflective affective evaluations (*d* = 1.22). AEE and reflective affective evaluations toward the fitness center setting were more positive for fitness center exercisers than in comparison group. Only nonsignificant correlations between AEE and reflective affective evaluations in center exercisers and comparison group.	First-grade
2	Antoniewicz and Brand, 2016b	88 exercise course participants, *M* = 24.98 (*SD* = 6.88)	Prospective, 14-week exercise program	Examine the impact of AEE for exercise course adherence	BIAT with exercise or nonexercise activities pictures and emoticons (good, bad)	**Proximal**: objective assessment of exercise adherence (resulting in: maintainer, early dropouts, late dropouts)	ANOVA showed that AEE were similar for the three exercise adherence groups at the beginning. A MANOVA revealed a significant group effect on positive and negative exercise associations (*d* = 0.54). Results of a *post hoc* discrimination analysis indicated that positive exercise associations contributed more to adherence group classification than negative associations.	Second-grade
3	Berry et al., 2011	53 under-graduate university students, *M* = 21.9 (*SD* = 5.4)	Cross-sectional	Examine differences in AEE in people with different exercise self-schema (exercisers, nonexercisers)	IAT with exercise pictures and evaluative words	**Proximal**: activity level (four groups of exercisers) **Distal**: exercise self-schema (exerciser, nonexerciser)	ANOVA and *post hoc* tests between exercise groups showed a marginally significant (*p* < 0.06) difference between AEE in the most active group (i.e., more positive AEE) and the groups with a lower exercise level (*d* = 0.81). ANOVA and *post hoc* tests revealed that AEE of “exerciser schematics” were significantly more positive than in “nonexerciser schematics” (*d* = 0.77).	First-grade
4	Bluemke et al., 2010	94 university students, *M* = 23 (*SD* = 3.3)	Cross-sectional	Investigate the relation between AEE and habitual exercise volumes/ short-range exercise behavior	EP with exercise-specific or generic verbs as prime stimuli and positive or negative exercise-specific or generic words as target stimuli	**Proximal**: exercise status: exercisers (nonsport students), exercisers (sport students), nonexercisers; habitual exercise behavior (frequency, duration and amount per week)	ANOVA indicated that both exerciser groups hold significantly more positive AEE than nonexercisers (*d* = 0.59). Ordinal regressions showed that AEE predicted self-reported frequencies of exercising, durations of typical exercise sessions and overall amounts of exercising per week.	First-grade
5	Brand and Antoniewicz, 2016	44 fitness club exercisers, *M* = 41.27 (*SD* = 14.06)	Cross-sectional	Examine the impact of automatic-reflective affective evaluation discrepancies on exercise behavior	ST-IAT with exercise pictures and emoticons (good, bad)	**Proximal**: aspired exercise behavior; actual exercise behavior; actual/ aspired exercise ratio **Distal**: reflective affective evaluations; automatic-reflective affective evaluation discrepancy (ARED) scores: sum (ARED_sum) and differences (ARED_diff) of AEE and reflective affective evaluation scores	AEE was significantly correlated with the actual/aspired exercise frequency ratio (*r* = 0.32). Multiple regression analyses showed that ARED_sum predicted the actual exercise frequency, *b* = 3.83 (*R*^2^ = 0.10). ARED_diff predicted the aspired exercise frequency, *b* = 4.74 (*R*^2^ = 0.10) and the actual/aspired frequency ratio, *b* = −0.10 (*R*^2^ = 0.12).	First-grade
6	Brand and Schweizer, 2015	74 university students; 36 women, *M* = 23.2 (*SD* = 3.8), 38 men, *M* = 26.1 (*SD* = 3.6)	Cross-sectional	Identify the impact of AEE, reflective (cognitive and affective) evaluations on situated decisions about exercising	EP with exercise-specific or generic words as prime stimuli and positive or negative exercise-specific words as target stimuli	**Proximal:** exercise amount **Distal:** situated decisions to exercise; reflective (cognitive and affective) evaluations	Path analyses revealed that AEE was not associated with the reflective evaluations. AEE significantly predicted situated decisions to exercise (β = 0.15) additionally to the reflective evaluations. The more positive the reflective evaluations and AEE, the more likely participants are to decide in favor of exercising in the face of a behavioral alternative. Together, AEE and reflective evaluations explained 61% of variance in the situational decisions on exercise variable, which in turn predicted duration of exercise per week.	First-grade
7	Calitri et al., 2009	125 students, *M* = 23 (*SD* = 6)	Cross-sectional	Relation of attention bias, automatic and reflective affective and cognitive evaluations, and exercise behavior	EAST with exercise and neutral/control words	**Proximal:** self-reported exercise behavior in the past week (type, frequency, intensity and duration); **Distal:** reflective affective evaluations; reflective cognitive evaluations; visual attention	Significant correlation between AEE and exercise behavior (*r* = 0.22). No reliable linear correlation between AEE and visual attention bias but results of curve estimation revealed a significant “u-shaped” quadratic relationship (β = 0.29). Additional moderated hierarchical regression analyses showed that AEE did not significantly moderate the association between attention and exercise behavior. Separate hierarchical multiple regression analysis revealed that reflective cognitive evaluations and reflective affective evaluations did not significantly interact with AEE in their association with previous exercise behavior. Sequential multiple regression showed that attention bias to exercise along with AEE are significantly associated with exercise behavior (*R*^2^ = 0.14).	First-grade
8	Chevance et al., 2016	59 obese participants, *M* = 34.7 (*SD* = 12.3) and 94 participants from the general population, *M* = 50.6 (*SD* = 8.9)	Cross-sectional	Investigate the additional contribution of AEE in the TPB framework	IAT	**Proximal:** physical activity and exercise behavior in the past week **Distal:** TPB variables	AEE was nonsignificantly correlated with physical activity and exercise behavior in the overall sample. Multiple regressions revealed that AEE (β = 0.25) additionally to TPB variables (β = 0.38) was a significant predictor of exercise behavior among obese people, but not in the general population.	First-grade
9	Craeynest et al., 2005	38 obese children and adolescents, *M* = 13.69 (*SD* = 2.63) and 38 normal-weight children and adolescents, *M* = 13.53 (*SD* = 2.52)	Cross-sectional	Identify differences of AEE in obese and normal-weight children	EAST with physical activity and exercise words for sedentary behavior, and activities in moderate and high intensity	**Distal:** Extrinsic response valence to exercise words (positive, negative)	A 2 (group) × 3 (exercise intensity) × 2 (word valence) ANOVA found no significant effects. Youngsters with obesity did not have more positive automatic evaluations toward sedentary activities. Neither did they have more pronounced negative AEE.	First-grade
10	Craeynest et al., 2008	19 obese children and adolescents, *M* = 12.79 (*SD* = 2.68)	Prospective; 1 year, T0: baseline during first 3 weeks of treatment, T1: + 6 months, T2: follow-up after 1 year	Examine the influence of AEE on obesity treatment results	EAST with physical activity and exercise words for sedentary behavior and activities in moderate and high intensity	**Distal:** extrinsic response valence to exercise (positive, negative); ABMI	A 3 (exercise intensity) × 2 (word valence) RM-ANOVA at baseline AEE showed no significant effects. A 3 (time) × 3 (exercise intensity) × 2 (word valence) RM-ANOVA only revealed a significant main effect of time, *F*_(2, 17)_ = 10.22. This indicates that participants reacted faster on persecuting tests. A standard multiple regression analysis showed that a change of AEE toward high-intensity exercise was a significant predictor of ABMI change after treatment (β = 0.51), adjusted for age, sex and baseline ABMI. Another multiple regression analysis revealed that only the change in AEE toward moderate exercise was a significant predictor of follow-up ABMI (β = 0.89), adjusted for age, sex and baseline ABMI.	First-grade
11	Endrighi et al., 2016	100 female patients previously diagnosed with endometrial cancer, *M* = 57.0 (*SD* = 11.01)	Prospective; 6 month, T0: baseline, T1: + 2 month, T2: + 4 month, T3: + 6 month	Examine the influence of AEE on exercise behavior in cancer survivors	IAT	**Proximal:** daily minutes of exercise **Distal:** reflective affective evaluations; exercise self-efficacy	Linear mixed models revealed that baseline AEE were not significantly associated with subsequent exercise self-efficacy changes after 2, 4 or 6 month. Baseline AEE were significantly predictive for self-efficacy changes when considering reflective affective evaluations and self-efficacy at baseline (*r* = 0.17). No significant associations emerged between baseline AEE and daily minutes of exercise after 2, 4 or 6 months.	Second-grade
12	Eves et al., 2007	188 Royal Air Force trainee aircraftsmen, *M* = 20.0 (*SD* = 3.7)	Cross-sectional	Examine the impact of AEE on walking and running behavior	EP with moderate and high-intensity exercise words as prime stimuli, and negative and positive exercise-unspecific evaluative words as target stimuli	**Proximal:** walking behavior (pedometer for 1 week); self-reported exercise behavior in the past week (frequency for moderate- and high-intensity activities as well as for walking and running); running in following week **Distal:** intention to run	For moderate- and high-intensity activities in the past week (running and walking excluded) a between factor ANOVA showed a significant main effect for word valence (good vs. bad, *d* = 0.49). Response latencies for the positive words were shorter. No differences between groups (high vs. low frequency of moderate- and high-intensity activities). There was a main effect for participation in running in the past week (*d* = 0.63). It was additionally shown that there was an interaction between participation in running in the next week and word valence (good vs. bad, *d* = 0.62). Those who did not run in the next week showed shorter response latencies for negative words and longer latencies for positive words.	First-grade
13	Karpen et al., 2012	134 undergraduate students, *M* = n.a. (*SD* = n.a.)	Cross-sectional	Examine the impact of IED toward exercise on changes in self-perception and reflective evaluations, by a self-perception manipulation	AMP with exercise equipment pictures or pictures of household items	**Distal:** reflective (affective and cognitive) evaluations; IED; self-perception of exercise importance	Multiple regression analysis showed that neither AEE nor reflective evaluations alone significantly predicted the change of self-perception or the change of reflective evaluations after a self-perception manipulation. There were effects for IED: self-perception (β = 0.43) and reflective evaluations (β = 0.35) of those with greater IED were significantly more strongly affected by self-perception manipulation.	First-grade
14	Sala et al., 2016	69 psychology students, *M* = 20.4 (*SD* = 2.4)	Cross-sectional	Investigate whether anticipated affective factors (e.g. AEE) are related to affective responses during and after exercise	SC-IAT with words representing exercise	**Distal:** affective response to exercise in moderate intensity (during, post-exercise)	Regression analysis revealed that AEE did not significantly predict affective response during exercise. Additionally, AEE did not significantly predict immediate post-exercise affective response.	First-grade

*ABMI, adjusted BMI; AEE, automatic evaluations of exercising; AMP, Affect Misattribution Procedure; ARED, automatic-reflective evaluation discrepancy; BIAT, Brief IAT; EAST, Extrinsic Affective Simon Task; IAT, Implicit Association Test; IED, implicit-explicit attitudinal discrepancy; EP, Evaluative Priming Task; TPB, Theory of Planned Behavior; SC-IAT, Single-Category IAT; ST-IAT, Single-Target IAT*.

**Table 2:** Experimental studies investigating automatic evaluations of exercising and exercise indicators (*k* = 6).

	**Study**	**Participants**	**Study design**	**Main aim**	**AEE measure**	**Main IV**	**Main DV**	**Overall findings concerning AEE**	**Grade of evidence rating**
1	Antoniewicz and Brand, 2016a	64 undergraduate sport students, *M* = 23.02 (*SD* = 2.44)	Experimental; **intervention**: EC, participants randomized to 1 of 3 groups: positive EC (*n* = 19), negative EC (*n* = 20) and control (*n* = 25)	Investigate the alterability of AEE	ST-IAT with exercise pictures and words related to feelings and bodily sensations	EC with exercise and nonexercise-related pictures (CS) and pictures of people displaying strong positive or negative feelings (US)	**Distal:** AEE	ANOVA showed a significant group effect on AEE (*d* = 0.70). Planned contrasts revealed significant differences between the positive EC group and control group (*d* = 0.77).	Third-grade
2	Antoniewicz and Brand, 2016a	41 female psychology students, *M* = 23.51 (*SD* = 4.36)	Quasi-experimental; **intervention**: EC, participants were assigned to 1 of 3 groups incongruent to their previously assessed AEE: positive EC (*n* = 13), negative EC (*n* = 14) and control (*n* = 14)	Examine the effect of altered AEE on subsequent exercise behavior	EP with exercise-specific or nonexercise words as prime stimuli and positive and negative exercise-specific evaluative words as target stimuli	EC with exercise and nonexercise-related pictures (CS) and pictures of people displaying strong positive or negative feelings (US)	**Proximal:** subsequent exercise behavior (chosen exercise intensity on bicycle ergometer)	ANOVA showed a significant group effect on choice of exercise intensity (*d* = 1.28). Planned contrasts revealed that the positive EC group selected significantly higher intensities than the control group (*d* = 0.88). No significant differences of selected intensities between the control group and the negative EC group.	Second-grade
3	Berry, 2016	155 first-year psychology students, *M* = 19.4 (*SD* = 1.96)	Quasi-experimental; **intervention**: reading targeted exercise information, participants were assigned to 1 of 5 groups according to their pretest reflective evaluations: negative affective (*n* = 27), positive affective (*n* = 21), negative cognitive (*n* = 36), positive cognitive (*n* = 16), control (*n* = 39)	Investigate the alterability of AEE	GNAT with exercise or generic words as target category and affective or cognitive evaluative words as evaluative category	Text with targeted exercise-related (1) negative affective, (2) positive affective, (3) negative cognitive, (4) positive cognitive information or a text about cooking (control)	**Distal:** AEE	RM ANOVA revealed a significant time × condition interaction (*d* = 0.67). *Post hoc* tests showed a significant positive within-subject change in AEE in the negative cognitive information condition (*d* = 0.71).	Second-grade
4	Berry et al., 2013	138 undergraduate psychology students, *M* = 20.4 (*SD* = 5.42)	Experimental; **intervention**: sequence from The Biggest Loser (BL) or American Idol (AI), participants randomized to 1 of 2 groups: BL (*n* = 63), AI (*n* = 64)	Investigate the alterability of AEE	GNAT with exercise or generic words as target category and affective words as evaluative category	Video clip with depiction of strenuous exercise (sequence from The Biggest Loser)	**Distal:** AEE; reflective affective evaluations; mood (POMS); thought-listing valence	RM ANCOVA showed no significant differences in AEE between groups. There was no significant within-subject effect. Neither mood nor activity level were significant covariates. Correlation between the thought-listing valence score and AEE was significant (*r* = 0.39). There was a significant correlation between AEE and reflective affective evaluations (*r* = 0.25).	Third-grade
5	Berry and Shields, 2014	213 undergraduate psychology students, M = 19.06 (*SD* = 1.78)	Experimental; **intervention**: health- or appearance-orientated exercise advertisement, participants randomized to 1 of 2 groups: health condition (*n* = 110), appearance condition (*n* = 103)	Investigate the alterability of AEE	GNAT with exercise or generic words as target category and affective or cognitive evaluative words as evaluative category	Video clip of exercise advertisement (health- or appearance-orientated)	**Distal:** AEE; automatic cognitive evaluations	MANCOVA revealed no significant main effect for video clip group. RM MANOVA showed a significant multivariate within-subject effect for differences between *d*' scores for good and bad automatic cognitive evaluations and AEE (*d* = 1.88). There were significant within-subject differences for automatic cognitive evaluations (*d* = 1.85), but not for AEE.	Third-grade
6	Markland et al., 2015	160 undergraduate and postgraduate students, *M* = 23.03 (*SD* = 4.83)	Experimental; **intervention**: imagery intervention exercise or control, participants randomized to 1 of 2 groups matched by gender and exercise status: exercise (*n* = 80), control (*n* = 80)	Investigate the alterability of AEE	IAT with exercise pictures and evaluative words	Guided imagery of a visit to a fitness facility or of preparing and eating a meal (control)	**Proximal:** exercise status **Distal:** AEE; reflective affective evaluations; reflective cognitive evaluations	There were significant correlations between AEE and reflective affective evaluations (*r* = 0.32), and AEE and reflective cognitive evaluations (*r* = 0.24). A two-factor ANOVA (imagery condition × exercise status) found a significant main effect for imagery condition (*d* = 0.41) and exercise status (*d* = 0.59). No significant interaction. AEE were more positive in exercise imagery group than control group (*d* = 0.39). Frequent exercisers had more positive AEE than less frequent exercisers (*d* = 0.57).	Second-grade

*AEE, automatic evaluations of exercising; CS, conditioned stimulus; DV, dependent variable; EC, Evaluative Conditioning; EP, Evaluative Priming Task; GNAT, Go/No-go Association Task; IAT, Implicit Association Test; IV, independent variable; POMS, Profile of Mood States; ST-IAT, Single-Target-IAT; US, unconditioned stimulus*.

## Results

**Figure 1:**
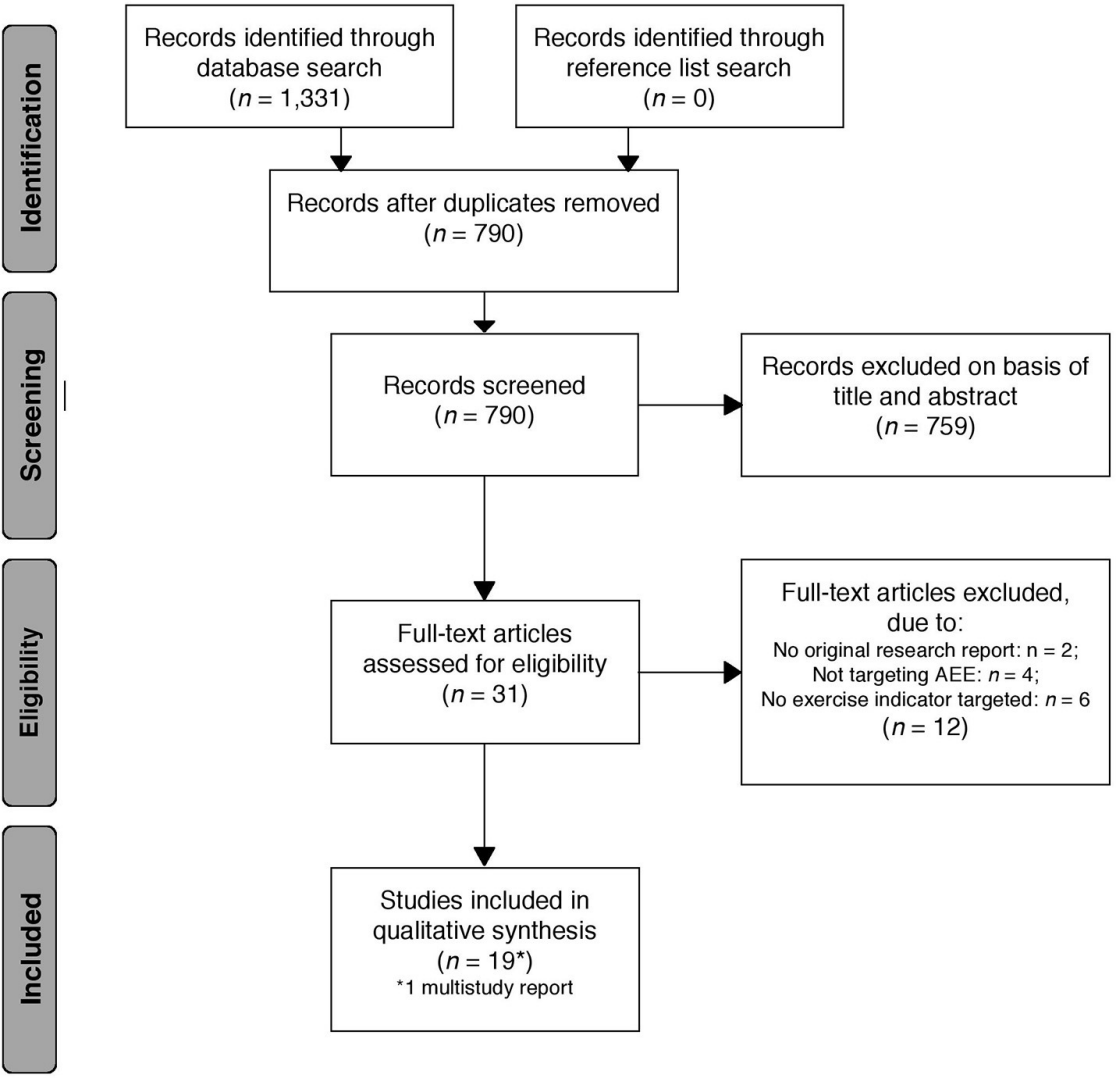
Study selection flow chart according to PRISMA statement (Moher et al., 2009).

The literature search identified a total of 1,331 records (see Figure 1 for PRISMA flow chart). No additional studies were identified through records' reference lists. After excluding duplicates and records not meeting the described eligibility criteria by checking the titles and abstracts, 31 records remained for full-text analysis of eligibility. In total, 19 records, including one that was a multi-study report of two separate studies, fulfilled the eligibility criteria and were included in this review. Of these, six studies had an experimental design and 14 were nonexperimental (cross-sectional or prospective).

### Study characteristics

#### Non-experimental studies

From the included nonexperimental studies, 11 were cross-sectional and a further three had a prospective design, ranging from 3.5 months to 1 year and three to four times the measurement. The overall sample size of all included nonexperimental studies was *N* = 1,157 participants, ranging from 19 to 188 participants per study. Studies either explored male and female participants (*k* = 13) or focused on female participants only (*k* = 1). The samples included university students (*k* = 7) or groups of center exercisers or exercise course participants (*k* = 2), aircraftsmen (*k* = 1), cancer patients (*k* = 1), or obese children and adolescents (*k* = 3; which were compared to normal-weight children and adolescents). Average age ranged from *M* = 12.79 (*SD* = 2.68) to *M* = 57.0 (*SD* = 11.01). Due to their nonexperimental design, the majority of studies were rated as first-grade evidence (*k* = 12). Two studies reached a second grade of evidence rating (see Tables 1, 2 for the rating of each study).

#### Experimental studies

All experimental studies investigated the effects of different interventions on the alteration of AEE (*k* = 6). One study additionally examined the effect of altered AEE on subsequent exercise behavior. The design of the interventions was experimental (*k* = 4) or quasi-experimental (*k* = 2), ranging from two to five experimental conditions. All studies applied a single-session intervention to alter AEE. Interventions were diverse, using Evaluative Conditioning (EC; see Hofmann et al., 2010 for an overview; *k* = 2), video clips depicting exercising individuals (*k* = 1) or an exercise advertisement (*k* = 1), a text with targeted exercise-related information (*k* = 1) or a guided imagery intervention (*k* = 1). The overall sample size was 771 participants, ranging from 41 to 213 participants per study. The participants comprised undergraduate psychology students (*k* = 4), undergraduate sport students (*k* = 1) or under- and postgraduate students of different subjects (*k* = 1). Average age ranged from *M* = 19.06 (*SD* = 1.96) to *M* = 23.51 (*SD* = 4.36). Studies included either male and female participants (*k* = 5) or investigated female participants only (*k* = 1). Three studies were rated as second-grade evidence and a further three studies were rated as third-grade evidence.

### AEE measurement

A first finding of this review concerns an immense diversity in the applied methods to measure AEE. Most of the identified studies used variants of the IAT (*k* = 7), which measure the strength of associations between semantic concepts stored in the memory. Standard IATs (*k* = 4; Berry et al., 2011; Markland et al., 2015; Chevance et al., 2016; Endrighi et al., 2016) are lexical sorting tasks in which stimuli from the target (e.g., “exercise”) vs. a comparison category (e.g., “nonexercise”) and from two evaluative categories (e.g., “good” vs. “bad”) are mapped to the same computer key. The sorting task is easier for respondents when the concepts sharing the same response key are closely associated than when they are not. A person with negative AEE will respond more quickly when, for example, the words “exercise” and “bad” are mapped to the same key than when “exercise” and “good” are mapped to the same key. IAT scores are usually calculated using the difference between response times for related and unrelated word pairs (Greenwald et al., 2003). Most IATs used pictures of exercise equipment or people engaging in exercise to represent the category “exercise.” Therefore the depicted exercises differed in their intensity within and between the studies (e.g., running on a treadmill, stretching, performing resistance exercise, using an exercise ball; Markland et al., 2015). Astonishingly, all studies used different comparison categories (e.g., “couch potato,” “inactivity” or “not exercise”) represented by pictures of diverse activities (e.g. reading a book, resting, relaxing, watching television).

Two studies (Antoniewicz and Brand, 2016a; Brand and Antoniewicz, 2016) used the Single-Target IAT (ST-IAT; Bluemke and Friese, 2008), one study (Sala et al., 2016) used the Single-Category IAT (SC-IAT; Karpinski and Steinman, 2006) and another one (Antoniewicz and Brand, 2014) used the Brief IAT (BIAT; Sriram and Greenwald, 2009). In comparison to the original IAT, the ST-IAT, SC-IAT and BIAT emphasize the strength of associations between one focal target category (e.g., “exercise”) and its evaluation (“good” or “bad”) so that the participants only have to pay attention to one concept. In addition, the BIAT is an abbreviated version of the IAT and is thus timesaving. The two ST-IAT studies used the same eight pictures to represent “exercise” (two different gymnastic exercises, strength training, running, swimming, volleyball, soccer, and tennis). For the SC-IAT, words representing exercise in different intensities were used. For the BIAT, diverse exercise-related pictures represented the “exercise” category. The BIAT study and one of the ST-IAT studies used symbols representing smiling or frowning faces (so-called “emoticons”) to depict the evaluative categories “good” and “bad.”

Three studies (Craeynest et al., 2005, 2008; Calitri et al., 2009) applied the Extrinsic Affective Simon Task (EAST; De Houwer, 2003) to measure AEE. The EAST is conceptually related to the IAT but differs procedurally. In comparison to the IAT, participants respond to evaluative target words written in white and (exercise-specific) target words written in different colors (e.g., blue and green) by pressing one of two computer keys. Participants have to respond to white attribute words in terms of their valence whereas they have to ignore (the irrelevant) valence for colored words and respond only in terms of color. For example, participants are asked to respond with one key for “good” and “blue words” and with another for “bad” and “green words.” Participants with positive AEE perform more quickly when the target color of the (colored) exercise word and positive valence are sorted under the same response key. This effect relies on the congruence of the task-irrelevant valence of the target word and valence response. The intensity of exercises represented by the exercise words was diverse, ranging from walking to rowing and sprinting. Four studies (Eves et al., 2007; Bluemke et al., 2010; Brand and Schweizer, 2015; Antoniewicz and Brand, 2016a) applied an EP task, which assesses automatic evaluative associations through a basic procedure of sequential priming (Fazio et al., 1986). In this task, a prime stimulus (e.g., exercise word or control word) is briefly presented and followed by a positive or negative word as target stimulus (e.g., “pleasant,” “delightful” vs. “repulsive,” “disgusting”). Participants are asked to categorize positive target words to one key and negative target words to another. It is assumed that the presentation of the prime activates associated evaluative attributes. For people with positive AEE, the response to positive target words after an exercise prime is thus facilitated (in comparison to a control prime) and leads to faster response times. All these studies used exercise words representing various exercise intensities and, as the comparison or control category, words representing nonexercise activities, nonsense words, or food words. Three studies (Bluemke et al., 2010; Brand and Schweizer, 2015; Antoniewicz and Brand, 2016a) explicitly used exercise-specific positive and negative words (e.g., “relaxed” vs. “exhausted”) as target stimuli.

Another five studies deployed implicit methods that do not rely on reaction time differences. The Go/No-go Association Task (GNAT; Nosek and Banaji, 2001) was applied in three studies (Berry et al., 2013; Berry and Shields, 2014; Berry, 2016). The GNAT is utilizable for the assessment of associations involving a single target category. Participants are asked to show a go response, by pressing a key, to target stimuli (e.g., exercise words and positive words) and a no-go response, by not pressing a key, to distracter stimuli (e.g., negative words). Unlike the IAT, differences in error rates (analyzed as sensitivity scores) of the responses to target words (e.g., “exercise”) indicate the strength of association between the target concept and a related evaluation. Positive AEE are assumed to ease the accuracy in discriminating “exercise” and “good” from distracters. All three GNAT studies assessed AEE with the target category “exercise” and another “generic” category. To represent “exercise,” diverse words were used (e.g., “workout,” “active,” “sports”). Two studies (Karpen et al., 2012; Antoniewicz and Brand, 2014) made use of the Affect Misattribution Procedure (AMP; Payne et al., 2005). The AMP applies a sequential priming procedure to assess affective associations through evaluative responses (more pleasant or less pleasant). This method differs from the previously mentioned methods in several ways. The evaluated stimulus is an ambivalent Chinese character, which has to be classified as “more pleasant” or “less pleasant” than an average Chinese character. Prior to the Chinese character, exercise pictures or pictures from a “neutral” category are presented as primes. The evoked affect is misattributed to the Chinese ideograph, which leads to shifts in the “more pleasant/less pleasant” ratings. Primes were shown supraliminally (*k* = 1) or subliminally (*k* = 1). Karpen et al. (2012) used images of household items as “neutral” primes whereas Antoniewicz and Brand (2014) used gray rectangles as control primes.

### AEE and associated exercise indicators

The identified studies examined hypotheses on the relation between AEE and exercise indicators and checked for AEE differences between different groups (see Tables 1, 2 for an overview). As for the applied methods, again, a respectable heterogeneity of the investigated exercise variables became apparent. The assessed exercise indicators can be categorized into three different domains. One group of studies addressed rather proximal exercise indicators, which directly represent quantitative (e.g., exercise volumes, exercise attendance) and qualitative (e.g., preferred exercise-setting) aspects of exercise behavior. A second group of studies targeted rather physiological or psychological variables that are associated with exercise behavior or decisions. These exercise indicators are summarized as distal exercise indicators (e.g., BMI changes, situated decisions to exercise or not to exercise). A third group of studies can be identified that assessed differences in AEE in specific target groups (e.g., obese individuals vs. normal-weight individuals).

#### Nonexperimental studies

##### Automatic evaluations of exercising and proximal exercise indicators

Within the nonexperimental studies targeting AEE and proximal exercise indicators, several studies focused on the differences between highly active and less active individuals. Bluemke et al. (2010) showed that “exercisers” hold more positive AEE than “nonexercisers” (*d* = 0.59). Likewise, Berry et al. (2011) found a marginally significant (*p* < 0.06) difference between AEE in highly active individuals (i.e., more positive AEE) and less active individuals (*d* = 0.81). Moreover, AEE were identified as predicting the self-reported frequency, the duration of typical exercise sessions and the amount of habitual exercise behavior per week. Calitri et al. (2009) revealed a significant, positive, small-sized correlation between AEE and self-reported exercise behavior in the past week (*r* = 0.22). The study of Eves et al. (2007) contributed to these findings as well and demonstrated that their participants' AEE were associated with running behavior in the following week (*d* = 0.63), with those not running in the subsequent week having negative AEE.

Among the proximal exercise indicators that have been investigated are qualitative aspects such as the preferred exercise-setting. Fitness center exercisers had more positive AEE of fitness center exercising than a likewise physically active comparison group that exercised at other settings (*d* = 0.59; Antoniewicz and Brand, 2014). Antoniewicz and Brand (2016b) assessed adherence to a 14-week exercise course not only on the quantitative level (i.e., amount of overall participation) but also on the qualitative level (i.e., consideration of individual participation patterns such as returning to the course after a missed session). The three resulting adherence groups (i.e., maintainers, early, and late dropouts) differed in their AEE (*d* = 0.54) already at the beginning of the exercise course and highlighted the predictive power of AEE for exercise adherence. Positive associations discriminated particularly well between later exercise course “maintainers,” “early dropouts” and “late dropouts” at the beginning of the exercise course.

Not all studies found the expected associations between AEE and proximal exercise indicators. In a longitudinal prospective study with female, previously diagnosed cancer patients, baseline AEE did not predict daily minutes of exercise after 2, 4, or 6 months. The authors argued that this lack of association might be due to generally positive AEE with limited variability in the study sample (Endrighi et al., 2016). Furthermore, AEE were not significantly associated with self-reported exercise behavior (Chevance et al., 2016) and self-reported frequency of moderate- and high-intensity activities in the past week (Eves et al., 2007).

##### Automatic evaluations of exercising and distal exercise indicators

Furthermore, distal exercise indicators and their association with AEE have been investigated. One of the first explorations of AEE showed that AEE influence people's visual attention to exercise cues (β = 0.29). Extremely negative and positive AEE led to elevated visual attention to exercise words (“U-shaped relation”; Calitri et al., 2009). Berry et al. (2011) targeted exercisers' self-schema and illustrated that people who identify themselves as “exercisers” had more positive AEE than “non-exerciser” schematics (*d* = 0.77). Brand and Schweizer (2015) showed that AEE influence situated decisions to exercise (β = 0.15). The more positive the AEE, the more likely people were to decide in favor of exercise in the face of a behavioral alternative. Additionally, it was shown that the tendency to decide for or against exercise predicts the habitual amount of exercise per week.

Chevance et al. (2016) showed that AEE (β = 0.25) incrementally predicted exercise behavior in obese adults over and above Theory of Planned Behavior (Ajzen, 1985; β = 0.38) variables. These findings were not present in the general population. The Theory of Planned Behavior, as one typical representative of the social-cognitive “information-processing paradigm” (Ekkekakis, 2017), involves reflective parameters like outcome expectancies and tries to explain intentions and resulting behavior from those variables. The authors concluded that AEE might be especially influential for obese individuals, which might be due to differences in self-regulation between obese and non-obese persons. Other studies explored correlations between AEE and reflective evaluations. Thereby some studies focused on reflective affective evaluations of exercise (e.g., “How pleasant is exercising in a fitness center for you?,” Antoniewicz and Brand, 2014) whereas others measured the relationship between AEE and cognitive components of reflective evaluations (e.g., “Exercising is: useless-useful, unnecessary-necessary, foolish-wise,” Karpen et al., 2012).

With regard to the association between AEE and reflective affective evaluations, Antoniewicz and Brand (2014) found no significant correlations in fitness center exercisers or the comparison group. Calitri et al. (2009) showed that neither reflective cognitive evaluations nor reflective affective evaluations interacted significantly with AEE in their association with self-reported exercise behavior in the past week. Moreover, AEE were not correlated with a combined measure of cognitive and affective components of reflective evaluations (Brand and Schweizer, 2015).

Some studies addressed discrepancies between AEE and reflective (affective and cognitive) evaluations of exercise. AEE and reflective evaluations alone did not significantly predict changes in self-perception and reflective evaluations after a self-perception manipulation. However, it was shown that self-beliefs (β = 0.43) and reflective evaluations of exercise (β = 0.35) were more strongly affected by a self-perception manipulation in individuals with larger discrepancies between AEE and reflective evaluations (Karpen et al., 2012). Brand and Antoniewicz (2016) advanced the idea of discrepant AEE and reflective affective evaluations. They developed combined scores for a more accurate description of the variable pairs' (AEE and reflective affective evaluation) sum and discrepancy. The sum of AEE and reflective affective evaluations predicted the actual exercise frequency of fitness club exercisers (*b* = 3.83, *R*^2^ = 0.10), whereas the discrepancy between AEE and reflective affective evaluations predicted the self-reported aspired exercise frequency per week (*b* = 4.74, *R*^2^ = 0.10) and the ratio of actual to aspired exercise frequency (*b* = −0.10, *R*^2^ = 0.12). Large discrepancies were associated with high self-reported aspired exercise frequencies. The authors interpreted these inflated goals as being a result of mistrust in the negative AEE and thereupon asserted very positive reflective affective evaluations (i.e., “idealized evaluations”; Brand and Antoniewicz, 2016). Low discrepancies predicted success in achieving the aspired exercise frequency. Also, AEE were positively associated with the ratio of actual to aspired exercise frequency (*r* = 0.32).

Not all expected associations between AEE and distal exercise indicators were found. For example, AEE neither predicted the affective response during moderate exercise nor the immediate post-exercise affective responses (Sala et al., 2016). Additionally, AEE were not associated with intention to run (Eves et al., 2007) or exercise self-efficacy (Endrighi et al., 2016).

Manifestations of AEE were also examined in specific target groups. Two studies focused on AEE and potential impact on obesity. They showed that obese youngsters neither had more negative AEE nor more positive automatic evaluations of sedentary behaviors than nonobese control individuals (Craeynest et al., 2005). A longitudinal study with obese children and adolescents in an obesity treatment setting found that favorable AEE of high-intensity exercise were a predictor of positive BMI change after obesity treatment (β = 0.51; Craeynest et al., 2008). Additionally, a within-person change in AEE of moderate-intensity exercise was a predictor of BMI change after 1 year of treatment (β = 0.89). A decrease in self-reported bodyweight was associated with increasingly negative AEE. Due to the small sample size (*n* = 19) and contextual inferences (some participants completed the EAST during treatment sessions, other at home or at university) these rather unexpected results should be interpreted with caution.

##### Automatic evaluations of exercising in specific target groups

Included studies assessed their data in diverse samples (e.g., students, general population), which also differed in age and gender. Two previously mentioned studies explicitly investigated the effects of AEE in specific samples. Eves et al. (2007) examined the effects of AEE on brisk walking (*p* > 0.05) and running (i.e., intention to run, *p* > 0.05; running behavior, *d* = 0.63) together with other moderate exercises (*p* > 0.05) in military trainee aircraftsmen. Another study used female previously diagnosed cancer patients as participants. There were no significant effects of baseline AEE on exercise self-efficacy changes or exercise behavior after 2, 4, and 6 months (Endrighi et al., 2016). Unlike the studies above (Craeynest et al., 2005, 2008) these studies did not compare specific samples to another sample (e.g., the general population). Due to the very specific populations the main study results should not be generalized.

##### Experimental studies

Experimental studies examined the possibility of altering AEE. Two studies used a computerized EC task (Antoniewicz and Brand, 2016a). In this task, pictures of exercising (conditioned stimuli) were repeatedly paired with positive or negative picture stimuli (unconditioned stimuli). The EC task resulted in differences in AEE between the group that should acquire positive AEE and the control group (*d* = 0.77) but not between the group intended to acquire negative AEE and the control group. Additional analyses revealed that changes in the group that acquired positive AEE were mainly driven by changes in associative connections between exercising and negative associations, in contrast to a facilitation of the associative connection between exercising and positive associations.

Markland et al. (2015) successfully altered AEE through a guided imagery intervention: AEE were more positive in the exercise imagery group (*d* = 0.39) than in a control group that imagined preparing a meal. Independently from the experimental manipulation, Markland et al. (2015) found that frequent exercisers had more positive AEE than less frequent exercisers (*d* = 0.57) and that AEE significantly correlated with reflective affective evaluations (*r* = 0.32) and reflective cognitive evaluations (*r* = 0.24).

In another study, participants read targeted exercise information that was incompatible with their pretest-AEE or pretest-reflective (affective and cognitive) evaluations of exercise. Results indicated that participants with positive AEE in the pretest who read information targeting negative reflective cognitive evaluations (e.g., information about negative health effects of exercise) unexpectedly showed even higher positive AEE in the posttest (*d* = 0.71; Berry, 2016).

Two other studies explored the alteration of AEE by using short video clips. One study used a short sequence from the TV show the biggest Loser, which depicted a strenuous exercise bout. There was neither a significant difference in AEE in comparison to a control group, nor a significant within-subject effect (Berry et al., 2013). In a study by Berry and Shields (2014), participants watched a short health- or appearance-orientated exercise advertisement. There were neither group differences nor significant within-subject differences in AEE.

To date, only one study has investigated how altered AEE affect subsequent exercise behavior. Antoniewicz and Brand (2016a) showed that AEE altered by EC influenced the choice of exercise intensity on a bicycle ergometer (main effect: *d* = 1.28). The group that learned positive AEE selected significantly higher intensities than the control group (*d* = 0.88). There were no significant differences in selected intensities between the group that should acquire negative AEE and the control group.

## Discussion

The main aim of this systematic review was to summarize and evaluate available research on AEE and exercising. Findings brought up small to large-sized correlations between AEE and exercise indicators in the vast majority of studies. Various implicit measures were used to assess AEE. Even when studies used the same measure, the specific application varied.

### Implicit measures to assess AEE

To the best of our knowledge, so far five different implicit measures have been used to assess AEE in exercise-related studies. Although very different measures have been used and the temptation is high to hand out advice that one might be the “best” implicit measure for assessing AEE, it is neither possible nor advisable to recommend “a particular paradigm as the best” (Gawronski and De Houwer, 2014, p. 293). In order to decide which measurement tool to use, every researcher has to answer the questions (among others) *what* exactly should be measured, as well as *how* and *when* it should be assessed. Since each measure has characteristic features, the fit between the respective research question as well as the underlying theoretical assumptions and the implicit measure's specific procedure is decisive. For all of these questions, the identified studies can provide some, albeit no complete, answers.

In the identified studies the predominant implicit measure is the IAT and variants of it (used in 40% of exercise-related studies). This finding corresponds with the dissemination of implicit measures in social cognition research in general where standard IATs are used in nearly every second published study (Nosek et al., 2011). As described before, standard IATs require two opposing categories, i.e., the target category and one for comparison. The studies in this review deployed “couch potato,” “inactivity” and “not exercise” in the classical IAT, which nicely illustrates the difficulty in finding a clear conceptual opposite of “exercise” (Rebar et al., 2015). In order to maximize the conceptual overlap between the implicit measure and the research design (Gawronski and De Houwer, 2014), researchers interested in AEE should check carefully whether their research questions include a comparison of “exercising” with another behavior. If not, other implicit measures might be more suitable for the respective research question. Only very few authors directly stated in their studies *why* a specific measurement procedure was used. Brand and Schweizer (2015) used an EP task and argued that the underlying mechanism (i.e., spontaneous evaluation of a stimulus) was closely related to the task requirements in their dependent variable (i.e., spontaneous decisions for or against exercising). The fit between research question and used method was thus explicitly taken into account. Antoniewicz and Brand (2014) targeted the automatic characteristic in AEE. They applied an AMP with subliminal stimulus presentation in order to conclude more easily on the automatic basis of AEE.

Exercising itself is a very complex and diverse behavior, which has to be reflected in AEE assessment. This systematic review has enumerated many different kinds of stimuli that have been used to assess AEE. Some of these stimuli represent the diversity more, some less. Again, the fit between the behavior of interest and the selected stimuli representing this behavior has to be considered. Some studies applied a very narrow focus and selected stimuli representing one specific behavior (e.g., fitness center exercising) in order to explain this tangible behavior (Antoniewicz and Brand, 2014), others used a broad range of stimuli in order to explain exercising behavior in general (e.g., Bluemke et al., 2010; Berry et al., 2011; Brand and Schweizer, 2015). A good indication of the selection of stimuli is the set provided by Rebar et al. (2016b). They aimed to establish population-level evidence of the most common exercise stimuli and ranked the 20 most-named activities when asked to report words relevant to the term “exercise.”

When measuring AEE and exercise indicators, it is essential to bear the affective nature of AEE in mind. This focus led to the exclusion of some studies that assessed automatic associations in the context of exercising but highlighted aspects other than the affective one (e.g., health; Berry et al., 2016, and exercise importance; Forrest et al., 2016). This differentiation is especially important when correlating AEE with reflective evaluations that measure more (see Calitri et al., 2009; Brand and Schweizer, 2015) or fewer (see Karpen et al., 2012) affective components.

Moreover, decisions about *how* to measure AEE should be guided by careful theoretical deliberations on the basis of dual-process theories. For the included studies, surprisingly, only a limited amount of studies explicitly referred to a dual-process theory in the theory section. Even less studies described relevant theoretical assumptions made by the stated dual-process theory (e.g., Bluemke et al., 2010; Berry et al., 2011) or, beyond that, directly explained how the theoretical assumptions guided decisions on an appropriate method to measure AEE (e.g., Brand and Antoniewicz, 2016).

The question *how* to measure AEE even includes one more issue. When the appropriate measure and stimuli are selected, the gained AEE scores can be calculated (even within the same measure) in different ways. Social cognition research is growing and progressing fast, which, for example, has led to alternative scoring algorithms for IATs. The established IAT *D*-Score has been extended to the DW-Score (Chevance et al., 2017b) and the IP-Score (Rebar et al., 2015), which have already been tested in the context of physical activity.

Lastly, the question *when* to measure AEE has been handled very differently in the identified studies. Prospective or retrospective assessments and mean scores resulting from the assessment before and after exercising have been used. While none of these approaches is right or wrong *per se*, the implications from each study vary widely. The retrospective assessment after an exercise bout could thus differ from prospectively assessed ones, due to the triggered automatic associations of exercising. Sound theoretical deliberations should thus guide the decision on the measurement point, in order to prevent a blending of findings, which can a priori be expected to be very different.

The (in)stability of automatic evaluations (Gawronski et al., 2017), in the context of physical activity (Hyde et al., 2012) and exercise (Antoniewicz and Brand, 2016a), has been much debated. Gawronski et al. (2017) demonstrated that automatic evaluations are less stable (weighted average *r* = 0.54) than conceptually corresponding reflective affective evaluations (weighted average *r* = 0.75). This finding can have methodological reasons, but can also be theoretically expected. For example, APE (Gawronski and Bodenhausen, 2011) points out that automatic evaluations are activated and altered on the mere basis of feature similarity and spatiotemporal contiguity whereas reflective evaluations are validated on the basis of logical consistency. Again, clarity on the underlying theoretical foundations of the study and on the measured construct can explain the assessment of sometimes more transitory or more long-lasting associations.

In sum, many different measures have been used to assess AEE. Quoting Nosek et al. (2011), the last few years of research can be characterized as the “Age of Measurement because of a proliferation of measurement methods and research evidence demonstrating their practical value for predicting human behavior” (p. 152). We claim to choose carefully between the available measures. They cannot be treated as substitutable and come with particular advantages and disadvantages that could fit or not fit with the research aim.

### AEE and exercise indicators

The current research was able to detect multiple associations between AEE and exercise indicators. As a first result of this systematic review, the examined exercise indicators were classified as proximal or distal in order to better systematize and evaluate the findings. Whereas, the applied proximal indicators are akin in some ways (e.g., assessment of exercise amounts), the distal exercise indicators we identified were very diverse, making it very difficult to relate the findings to each other.

#### Proximal exercise indicators

Quantitative aspects of exercising, such as the amount of exercise per week, have been examined repeatedly. In particular, the association between differently pronounced AEE in individuals with higher or lower exercise amounts has been confirmed in a number of studies. For the studies with similar exercise indicators, the seemingly comparable results need to be critically evaluated. In general, the literature underscores that frequent exercisers hold more positive AEE than less frequent exercisers and highlights the predictive power of these AEE. However, only a very limited number of studies used objective measures such as accelerometers to collect actual behavioral data. Since self-reports are susceptible to overreporting and recall bias (Duncan et al., 2001) and could be biased by specific self-concepts (especially when referring to exercising; Brewer et al., 1993), the collected data might not represent the actually executed exercise behavior. Although this inadequacy represents a systematic error within each study that does not necessarily influence the targeted association with AEE, the external validity and the comparability with other studies are limited. Using a more objective measurement for exercise behavior should become the rule rather than the exception in order to improve the precision and accuracy in future AEE research.

Taking a closer look at the different exercise intervals that have been associated with AEE, huge differences become apparent. While Antoniewicz and Brand (2016a) successfully demonstrated immediate behavioral differences after altering AEE, some studies used a time frame of 1 week (e.g., Calitri et al., 2009) and others referred to three- (Antoniewicz and Brand, 2016b) or 6-month periods (Endrighi et al., 2016). Even though not all of these studies detected the generally found significant relation between AEE and exercise behavior (Endrighi et al., 2016), AEE seem to be linked to both short- and long-term exercise behavior. These findings correspond with results from other research areas where automatic evaluations have successfully been used to alter immediate food choices (Hollands et al., 2011) or to explain weight gain over a year (Nederkoorn et al., 2010). However, due to the limited number of longitudinal studies included in this review the question whether AEE are better suited to predicting short- or long-term behavior can and should not be answered on the basis of current empirical evidence. Nevertheless, reflections on theoretical assumptions of dual-process theories can help to become an insight about the possible impact of AEE for long- and short-term exercise behavior. For example, in the APE model (Gawronski and Bodenhausen, 2011), AEE provide an evaluative basis (default-interventionist conception) for behavior. This could, on the one hand, explain the short-term approach and help to understand immediate decisions for or against exercising (Brand and Schweizer, 2015). On the other hand, the conception does not exclude the assumption that AEE can be associated with habitual or long-term behavior. Long-term behavior can be understood as repeated decisions to engage in exercise, which are, as this review has demonstrated, influenced by AEE. Repeated exercise experiences, such as exercising with a pleasurable feeling connected to it, can, according to learning theory, be understood as positive reinforcement that gradually changes or manifests AEE (Strack and Deutsch, 2004) and thus facilitates exercising.

A further point of discussion is the generally gained knowledge from most of the studies that assessed proximal exercise indicators. With the help of these studies we know *how* AEE differ between, for example, exercisers and nonexercisers. However, we do not know *why* AEE differ or under *which* conditions they are decisive for behavior execution. Another interesting question might be whether AEE vary or fluctuate among individuals whose exercise behavior is similar. Although the empirical findings do not yet provide answers to these questions, dual-process theories offer a wide range of explanations. Again, the APE model as an example (Gawronski and Bodenhausen, 2011) outlines specific operating principles and conditions for both processes. The operating conditions, for example, comprise issues such as intentionality, awareness, efficiency and controllability (the “four horsemen” of automaticity; Bargh, 1994) and describe exemplarily *how* the formation and expression of AEE takes place.

Another dual-process theory, the recently presented Affective-Reflective Theory (ART) of physical inactivity and exercise (Brand and Ekkekakis, 2017), describes under *which* conditions AEE have more or less impact on exercise behavior. One strength of ART is the explicit reference to the phenomenon of exercising and the unique bodily sensations related to it, which result in more or less pleasurable or displeasurable states during exercise. In the light of this theory, AEE are linked with action impulses of approaching or avoiding the bodily sensations associated with the behavior. As an operating condition, Brand and Ekkekakis (2017) designate the availability of self-control resources. Limited self-control resources (for an overview on the concept of self-control see Baumeister et al., 1998) would thus lead to a greater impact of AEE on exercise behavior. Some empirical findings might hint at this connection and outline the difficulties of individuals in adhering to an exercise regimen on days when self-control is lowered (Englert and Rummel, 2016), which could increase the impact of AEE. Additionally, it can be assumed that individuals with negative AEE require higher amounts of self-control in order to reach the same exercise behavior (e.g., amount of exercise per week) as those individuals with more positive AEE. Whereas, the exercise behavior is identical, the need to overcome negative AEE and corresponding behavioral schemata of avoiding is debilitating and may hinder to maintain the behavior over a longer period.

#### Distal exercise indicators

Distal exercise indicators comprise a great variety of variables (see Tables 1, 2). Among these, reflective affective evaluations and their interaction with AEE have been examined several times. Thereby studies either focused on correlations (e.g., Antoniewicz and Brand, 2014) or on the individual predictive power of AEE and reflective affective or cognitive evaluations (e.g., Karpen et al., 2012). Three studies did not find significant associations between AEE and reflective affective and/or cognitive evaluations (Calitri et al., 2009; Antoniewicz and Brand, 2014; Brand and Schweizer, 2015) while two studies found small-to-medium-sized associations between AEE and reflective affective and/or cognitive evaluations (Berry et al., 2013; Markland et al., 2015). The ambiguous findings can be explained by theoretical deliberations. As already mentioned dual-process theories postulate an interplay between AEE and reflective affective evaluations. Those interactions are, according to RIM (Strack and Deutsch, 2004) influenced by cognitive capacity, motivation, and the amount of attention. The APE posits that the interplay of automatic and reflective evaluations is mainly driven by the consistency or inconsistency of both evaluations (Gawronski and Bodenhausen, 2011). Further research is needed to evaluate the theoretical assumptions and examine *how* and under *which* conditions AEE and reflective evaluations interact.

Most of the studies assessed AEE and reflective affective evaluations distinctly. While this approach is necessary in order to get a better understanding of AEE and its unique impact on exercise behavior, it does not necessarily correspond to the theoretical assumptions of dual-process theories. According to dual-process theories, behavioral decisions are the result of interactions between both processes and thus between AEE and reflective affective evaluations (Strack and Deutsch, 2004; Gawronski and Bodenhausen, 2006). For example, according to APE, this interplay can either be described as a “bottom-up” influence (see Gawronski and Bodenhausen, 2011), in which automatic evaluations influence reflective evaluations, or as a “top-down” influence, which operates in the opposite way. One possible “bottom-up” influence could occur in the case of inconsistency between evoked AEE and consciously validated reflective affective evaluations. In order to avoid aversive feelings due to the resulting cognitive dissonance (Festinger, 1957), reflective affective evaluations would be adjusted. These kinds of adjustments should be taken into account when designing a study. Brand and Antoniewicz (2016) directly referred to this default-interventionist rationale and conducted a computerized sequential assessment of AEE and reflective affective evaluations, yielding dependent values for the two constructs and thus taking into account this pair of evaluations' temporal and functional relationship. “Top-down” influences can be characterized by processes of affirmation or negation. Negating the reflective evaluation “I dislike exercising” might strengthen the associative connection between “exercise” and “dislike” and translate into respective AEE.

In reference to ART (Brand and Ekkekakis, 2017), AEE and reflective affective evaluations can be understood to interact through reciprocal feedback. Those feedback loops are a prerequisite for learning and again highlight the default-interventionist connection between the two evaluations. Empirical examinations of the described mechanisms (such as by Berry, 2016) could help in understanding *why* AEE (and reflective affective evaluations) differ in some individuals and not in others.

In summary, the described state of evidence highlights the relevance of AEE for predicting proximal as well as distal exercise indicators. We want to emphasize that proximal and distal exercise indicators are very different things. Measuring people's intentions or exercise-related self-efficacy can and should not be mixed up with exercising behavior itself. It has to be treated as a variable that can, but does not necessarily have to, lead to exercising.

### AEE should not yet be targeted in exercise interventions

Some of the presented studies provide initial insights to the potential of targeting AEE in future exercise interventions (e.g., Calitri et al., 2009; Antoniewicz and Brand, 2014; Endrighi et al., 2016). However, before targeting AEE in exercise interventions, there has to be clarity on the causal connection between AEE and exercise behavior. Only six out of the 20 identified studies employed an experimental design and only one study addressed the AEE-exercise behavior link (Antoniewicz and Brand, 2016a), which would suggest a causal relation between AEE and exercising.

The general accessibility of AEE for interventions was addressed in five studies. These studies used different approaches to test the alterability of AEE: Either a theoretically driven or a more application-oriented approach was used. The theoretically driven approach (Antoniewicz and Brand, 2016a) used an EC task that, based on reflections of the formation of AEE in the APE model (Gawronski and Bodenhausen, 2006), systematically paired pictures of exercising (or nonexercising) with pictures eliciting positive or negative sensations. The encouraging result for possible interventions was the significant shift in AEE in the group acquiring positive AEE. Changes toward more negative AEE were not detected. In contrast to this approach, other experiments used materials such as advertisements or sequences from TV shows that might change AEE. Although the overall results are not consistent (e.g., Berry et al., 2013), an alterability of AEE by some of these techniques seems to be confirmed. Even though all experimental studies applied single-session interventions, they vary widely concerning contextual, procedural and temporal aspects. Whereas, the shortest manipulation included the presentation of 85 s long video clips (Berry and Shields, 2014), other manipulations took the participants several minutes to work on (Berry et al., 2013; Antoniewicz and Brand, 2016a). Longer manipulations, which might provide a bigger amount of associative learning possibilities, do not seem to be more successful (Berry et al., 2013) than rather short manipulations (Berry and Shields, 2014). Successful alterations of AEE could rather be due to the high density of the provided information (Antoniewicz and Brand, 2016a) or the personal involvement of the participants (Markland et al., 2015) in those studies. Replications of the described experiments may help to differentiate between effective and rather ineffective manipulations. It is important to note that there is no empirical evidence on the stability of the achieved changes. As the creation or modulation of associative links is based on the principle of contiguity, it is arguable how long-lasting the effects of these single-session interventions will be. Experimental designs that observe the sustainability of the manipulation effects or test the theoretical assumption that often co-activated mental representations lead to stronger AEE then singularly experienced contiguities, could serve as a basis for further deliberation about application in practice. More experimental evidence is urgently needed before we can ask for practical applications of the so far limited knowledge. The lack of research becomes particularly clear if one considers that only one experiment targeted the AEE-exercise behavior link. Antoniewicz and Brand (2016a) demonstrated that changes in AEE are connected to changes in actual exercise behavior. However, it is essential first of all to better understand what we are actually measuring with the implicit measures and what impact it has on the psychological system before considering whether, and how, we want to change that.

### Unresolved issues

This review provides the first systematic overview on the relation between AEE and exercise. While some questions concerning the relation can be answered, it is important to note limitations that require further research.

First, it should not go unmentioned that the results may represent a publication bias due to the preferential publication of statistically significant results in the last few decades. It has been noted that the selective publication of significant results represents a great threat to validity for meta-analyses and systematic reviews since the “published literature is systematically unrepresentative of the population of completed studies” (Rothstein et al., 2006, p. 1).

Second, the diverging use of related terms concerning the examined psychological construct (e.g., implicit vs. automatic vs. associative or attitudes vs. evaluations) and the inadequate distinction concerning the observed behavioral phenomenon (e.g., physical activity vs. exercise vs. sport) might have hindered the identification of all studies targeting the relation between AEE and exercise. In order to collect all studies fitting to our aimed-at research question, a large variety of (partly synonymous terms) was used. In the future, the extent of the use of different terms when referring to the same psychological construct should be reconsidered in order to avoid misunderstandings.

Third, the number of studies that achieved a grade-three evidence rating is very low (*k* = 3; 15% of all identified studies). This was predominantly driven by the considerable amount of studies that applied correlational designs. Correlational studies qualify for many differentiated conclusions and are doubtlessly necessary when starting to explore a new research area. However, in order to examine and understand the cause-effect relation, experimental designs are required. In general, the research field would profit from more experimental studies that target the mechanisms explaining the link between AEE and exercising and allow a causal connection between the two to be inferred.

Fourth, we started this review with the statement that AEE might help to understand the paradox phenomenon of nonexercising (despite the individual's reflective evaluation that exercising is e.g., healthy). Whereas, the review offers first insights on the relation between reflective evaluation and AEE (e.g., Calitri et al., 2009; Antoniewicz and Brand, 2014) and their respective impact on exercise decision and behavior (e.g., Brand and Schweizer, 2015), we want to point out that exercising and physical activity are two distinct behaviors that might be influenced by unique motivational factors (Biddle, 2011). Concluding from the provided findings on AEE and exercise behavior on the consequences for physical inactivity might be a shortsighted approach. None of the described studies directly assessed automatic evaluations of physical inactivity. We are aware of only one very recent study (Chevance et al., 2017a) employing two different SC-IATs to assess both, AEE and automatic evaluations toward sedentary behavior in obese individuals. They revealed that only AEE were related to exercise behavior, whereas automatic evaluations toward sedentary behavior did not predict exercising. These findings underline the necessity to understand exercising and sedentary behavior as two distinct behaviors with different motivational antecedents.

## Conclusion

As a result of our systematic review, we conclude that AEE are relevant determinants of exercise behavior, and are deserving much more research attention than they are actually given. Far-reaching conclusions are difficult to draw because of the immense heterogeneity concerning the observed exercise indicators, the implicit measures used to assess AEE and the underlying dual-process theories. This is tolerable, bearing the early phase of AAE research in sport and exercise psychology in mind. However, we claim that this review and the concomitant reassurance of the empirical evidence should mark the end point of this explorative phase of research. In order to achieve progress, a revision of the theoretical basis is urgently needed. We previously referred to Ekkekakis (2017), who diagnosed exercise psychology as being in a meta-theoretical crisis. In order to accelerate the transition from the “information processing paradigm” to dual-process theoretical frameworks, dual-process theories have to be scrutinized more thoroughly. Hence, it is an immense deficit that only a limited number of studies sufficiently described the underlying theoretical deliberations. Moreover, short-sighted, selected imports of automatic variables into existing theories from the “information processing paradigm” (for example, adopting the IAT for the add-on measurement of implicit attitude within the framework of the Theory of Planned Behavior) might not go far enough and might thus not reflect the explanatory potential of dual-process theorizing. Before handpicking and integrating single parameters into established theories, more empirical evidence on the coherence and principles of the operation of automaticity is required. Consequently, as long as all these preconditions are not fulfilled, wide-ranging implications for exercise interventions should be postponed. Yet in sum, we fully agree that AEE constitute a worthwhile target for further basic research that might in the future, according to the findings, lead to practical implications.

## Author contributions

MS contributed to the conception and design of the review and performed the initial literature search. MS and FA independently rated the studies and extracted the key variables. MS extracted the effect-sizes and transformed them when necessary. All passages of the review have been written mutually by both authors.

### Conflict of interest statement

The authors declare that the research was conducted in the absence of any commercial or financial relationships that could be construed as a potential conflict of interest.

## Footnotes

^1^The following search terms were used to identify the relevant articles: i. Exercis^*^ OR Sport^*^ OR physical activit^*^, ii. AND implicit OR automatic OR unconscious OR non-conscious OR associative OR impulsive AND, iii. Attitude^*^ OR Evaluation^*^/Process^*^ OR Cognition^*^/Attitude^*^ OR Evaluation^*^ AND “Dual process theor^*^”/Attitude^*^ OR Evaluation^*^AND “Dual process model^*^”/Attitude^*^ OR Evaluation^*^AND Process^*^ OR Cognition^*^.
